# Evaluation of outcome of acoustic reflex tests in patients with type 2 diabetes mellitus: a cross-sectional study

**DOI:** 10.1007/s00405-023-08065-y

**Published:** 2023-06-26

**Authors:** Puvvula Praneetha, Deviprasad Dosemane, Meera Niranjan Khadilkar, Kaushlendra Kumar, Anupriya Ebenezer

**Affiliations:** 1 Department of Otorhinolaryngology and Head and Neck Surgery, Kasturba Medical College, Mangalore, Manipal Academy of Higher Education, Manipal, 575001 India; 2 Department of Audiology and Speech Language Pathology, Kasturba Medical College, Mangalore, Manipal Academy of Higher Education, Manipal, 575001 India

**Keywords:** Acoustic reflex, Auditory pathways, Health, Pure tone audiometry, Type 2 diabetes mellitus

## Abstract

**Purpose:**

Type 2 diabetes mellitus (T2DM) may induce micro-vascular and macro-vascular changes that can lead to neuropathic changes which may affect the auditory pathway resulting in hearing loss. The study aims to evaluate the outcome of ipsilateral and contralateral acoustic reflex (AR) parameters and reflex decay tests (RDT) in patients with T2DM, and the relationship between average AR parameters, and duration and control of T2DM.

**Methods:**

An analytical cross-sectional study was conducted in a tertiary care setup in 126 subjects which included 42 subjects with T2DM between 30 and 60 years of age, age-matched with 84 non-diabetic subjects. The subjects were evaluated for pure tone average (PTA), speech identification score (SIS), AR parameters [acoustic reflex threshold (ART), acoustic reflex amplitude (ARA), acoustic reflex latency (ARL)] and RDT.

**Results:**

The subjects with T2DM showed increased PTA in both ears when compared to the subjects with no disease. No significant difference was found in the SIS between both groups. There was no significant difference in the ART and ARL between the two groups. There was a significant difference in the ipsilateral and contralateral ARA at 500 Hz, 1000 Hz and broadband noise (BBN) when compared between the diabetic and non-diabetic groups. No significant difference was found between average AR parameters and duration and control of T2DM.

**Conclusion:**

T2DM increases hearing thresholds and reduces ipsilateral and contralateral AR at lower frequencies and BBN. Duration and control of T2DM do not affect the AR parameters.

## Purpose

Diabetes mellitus (DM) is a chronic health disorder with hyperglycaemia resulting from insulin resistance and/or insulin inadequacy. The atlas of the International Diabetes Federation estimated that 10.5% of the world’s population is currently living with DM [1]. Type 2 diabetes mellitus (T2DM) may lead to macro- and micro-vascular changes such as thickening of capillary basement membrane, loss of inner hair cells and outer hair cells (OHCs), atrophy of spiral ganglion cells and marginal cells, and oedematous changes of the intermediate cells [2]. It may also lead to neuropathic changes and hearing loss due to the effect on the auditory pathway. Various research projects have identified the association between diabetes and hearing loss (HL) and discovered a positive correlation between the two variables [3–5]. The auditory system consists of complex but well integrated afferent and efferent pathways, with feedback circuits from the primary level to the associated auditory cortex. The high metabolic activity of the auditory pathway mandates adequate glucose levels, deeming it vulnerable to small glycaemic changes, thus affecting normal functioning. Prolonged hyperglycaemia may damage neural auditory pathways [6].

Our study intends to discover the influence of diabetes on the auditory system in diabetics by measuring acoustic reflex (AR) parameters and the reflex decay test (RDT), and also the link between the AR parameters and RDT with duration and control of diabetes.

## Methods

An analytical cross-sectional study was done in our tertiary care setup which included 42 subjects with T2DM (case group) aged between 30 and 60 years. This age range was chosen as the cut-off since age-related hearing loss is more common after 60 years and T2DM is known to often affect people after age 40. Age-matched 84 non-diabetic subjects (control group) who presented to the outpatient department with no ear complaints were assessed. Subjects with chronic middle ear disease, ear trauma, noise exposure, history of ototoxicity, facial nerve palsy, history of middle ear surgery, and familial history of congenital deafness were excluded. Subjects were selected by a non-random convenience sampling method. Basic details such as ear complaints, family history, duration, and treatment (with oral hypoglycaemic agents or insulin or both) of DM, random blood sugar and glycosylated haemoglobin (HbA1c) levels (up to 6.5% = normal, 6.6–8% = fair control and > 8% = poor control) were noted, followed by a thorough examination of ear, nose, and throat. Pure tone audiometry, tympanometry, speech identification scores (SIS), acoustic reflex tests (ART, ARL, ARA) and reflex decay tests (RDT) were performed on all the subjects. Pure tone audiometry was performed at various frequencies, i.e. 250, 500, 1000, 2000, 4000, and 8000 Hz and subjects with pure tone averages (PTA) < 25-decibel hearing loss (dBHL) were included in the study. Acoustic reflex threshold (ART) is defined as the lowest intensity at which acoustic reflex is elicited at each frequency and the acoustic reflex amplitude is explained as the maximum displacement of acoustic reflex for a given frequency. Acoustic reflex latency (ARL) describes the temporal characteristic of an acoustic reflex which illustrates the time course of the reflex. The reflex decay test (RDT) is defined as the estimation of the time at which reflex amplitude is 50% of the maximum amplitude [7]. According to ANSI (American National Standards Institute, 1982), the temporal characters of ARL are measured as initial latency (10% on) is defined as the time (in sec) from the beginning of an instantaneous immittance change to 10% of the measured steady-state immittance change, rise time (90% on) is defined as the time (in sec) from 10 to 90% of the measured steady-state immittance change, terminal latency (90% off) is defined as the time (in sec) from instantaneous termination of the initial immittance change to 90% of the measured steady-state immittance change and fall time (10% off) is defined as the time (in sec) from 90 to 10% of the measured steady-state immittance change after the initial immittance change is terminated [8]. AR parameters were evaluated at 500, 1000, and 2000 Hz and broad band noise (BBN). Approval was gained from the Institutional Ethics Committee (IECKMCMLR-09/2020/260) to conduct the study.

### Statistical analysis

Data collected were subjected to analysis using IBM SPSS Statistics for Windows, Version 25.0. Armonk, NY: IBM Corp. The variables were compared in proportions and mean (standard deviation; SD). *P* value of ≤ 0.05 was considered statistically significant.

## Results

The study included 126 individuals, with 42 cases and 84 controls. The case group included 23 (54.8%) males and 19 (45.2%) females and in the control group, there were 49 (58.3%) males and 35 females (41.7%).

An independent sample *t* test was performed to compare mean PTA and SIS between case and control groups. All 126 subjects showed PTA within normal limits, i.e., < 25dBHL; however, the case group showed increased PTA which was statistically significant in both ears (*p* value = right < 0.042, left < 0.001) when compared with the control group. No significant difference was noted in the mean SIS scores between both groups (*p* value right = 0.159, left = 0.689). All the subjects had an ‘A’ type tympanogram.

**Fig. 1:**
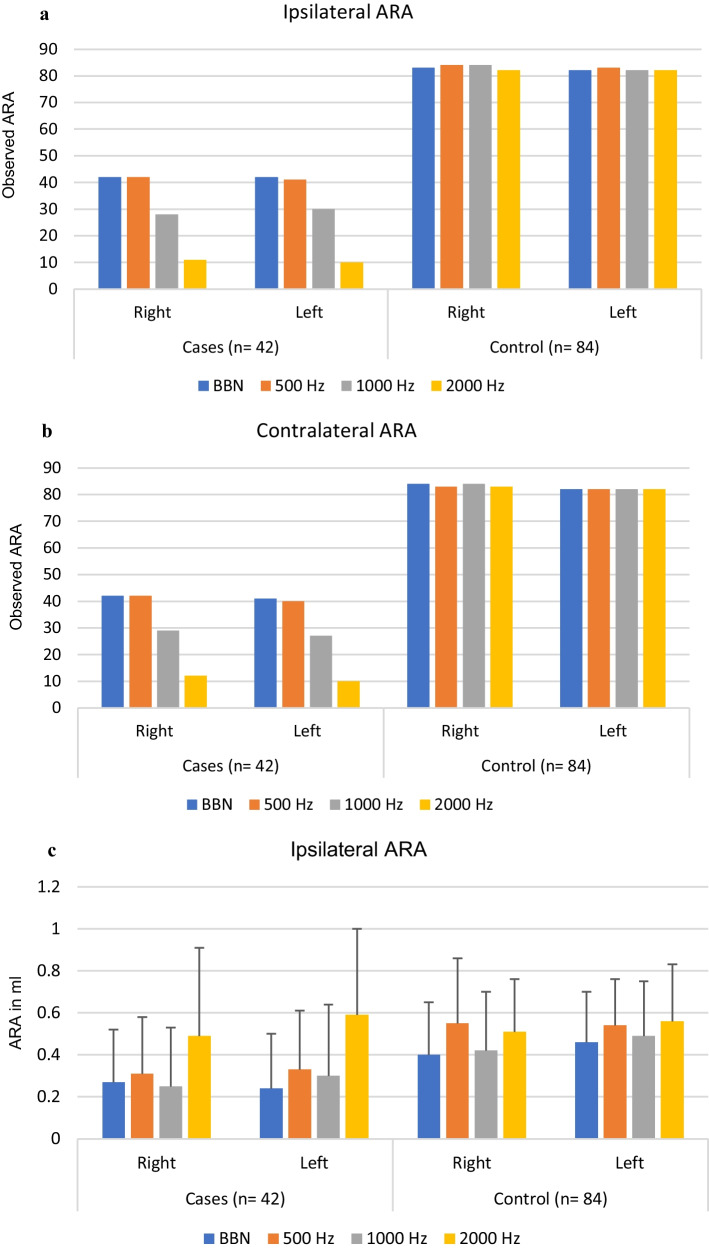

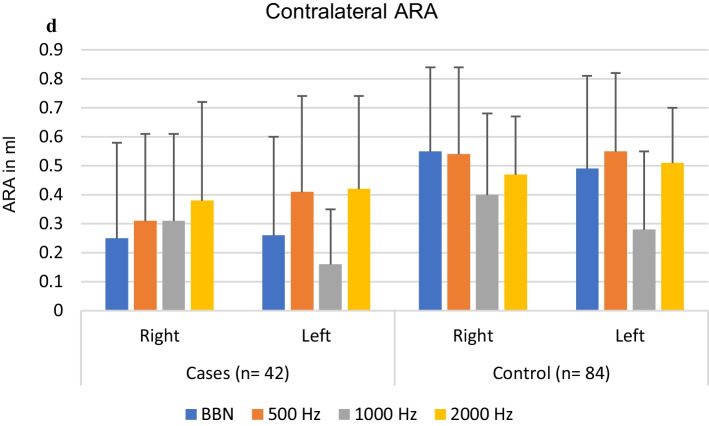
**a** Comparison between the observed ipsilateral right and left ARA in cases and controls. **b** Comparison between the observed contralateral right and left ARA in cases and controls. **c** Descriptive analysis (mean and SD) of ipsilateral ARA in both groups. **d** Descriptive analysis (mean and SD) of contralateral ARA in both groups

An independent sample *t* test was performed to compare ipsilateral and contralateral mean ART and ARA between cases and controls at 500, 1000, 2000 Hz and BBN. No significant difference was noted in the ipsilateral and contralateral mean ART between cases and controls. However, alterations of ARA (decreased amplitude) have been observed in the case group. There were a reduced number of reflexes (both ipsilateral and contralateral) in the case group when compared to the control group (Fig. 1a, b) and a significant difference was found in ARA at 500, 1000 Hz and BBN when compared between cases and controls (Fig. 1c, d).

**Fig. 2:**
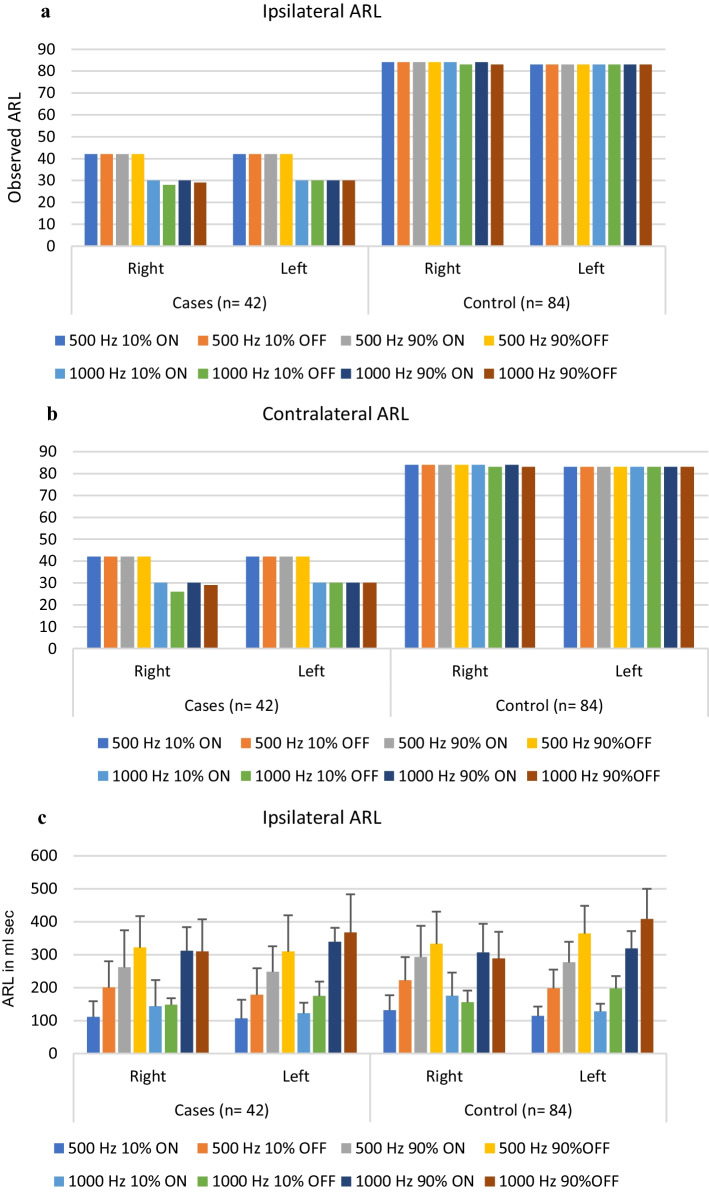

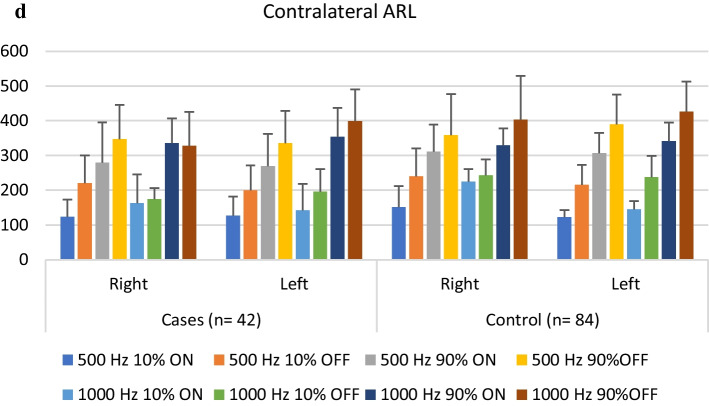
**a** Comparison between the observed ipsilateral right and left ARL in cases and controls. **b** Comparison between the observed contralateral right and left ARL in cases and controls. **c** Descriptive analysis (mean and SD) of ipsilateral ARL in both groups. **d** Descriptive analysis (mean and SD) of contralateral ARL in both groups

An independent sample *t* test was done to compare ARL between case and control groups at 500 and 1000 Hz. There was a significant difference in ARL in cases at 500, 1000 Hz (ipsilateral right 10% on) and at 500 Hz (ipsilateral left 90% on and left 90% off) and 1000 Hz (ipsilateral left 90% on and left 10% off) when compared with the control group (Fig. 2a–d). However, no significant difference was found between contralateral ARL (right and left) in both groups. All the subjects included in the study had no decay in the reflex decay test.

**Fig. 3:**
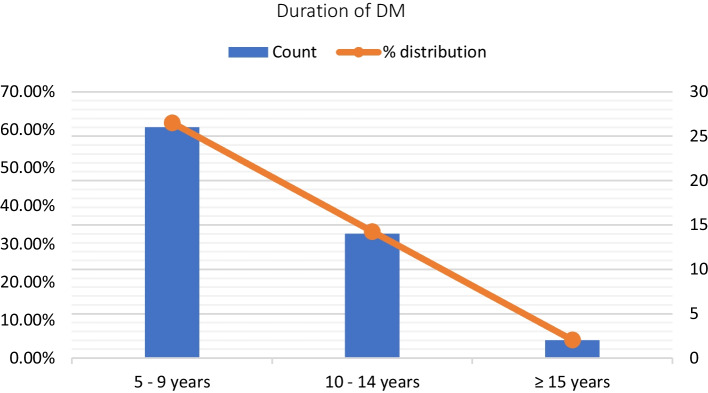
Subgroup Division of cases depicting their distribution based on the duration of diabetes mellitus (DM)

**Fig. 4:**
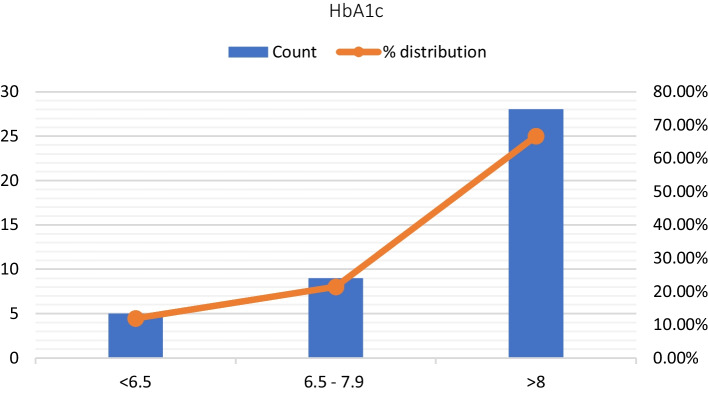
Subgroup Division of cases depicting their distribution based on control of DM with glycosylated haemoglobin (HbA1c) levels

One-way ANOVA test was performed to analyse the AR parameters and reflex decay tests in the case group based on the duration of T2DM and control of DM using HbA1c levels. The case group was subdivided into 3 groups; 5–9 years (61.9%), 10–14 years (33.3%) and ≥ 15 years (4.8%) based on the duration of diabetes (Fig. 3). Based on HbA1c levels, the case group was categorised into normal (HbA1c < 6 = 11.9%), fair control (HbA1c 6.5–7.9 = 21.4%) and poor control (HbA1c > 8 = 66.7%) (Fig. 4). Subgroup analysis was done; however, there was no significant difference in the AR parameter within the cases based on the duration and control of diabetes (Table 1, 2).

**Table 1:** Subgroup analysis of AR parameters in comparison with control of DM based on HbA1c levels (ANOVA analysis)

Tests	Variables (Hz)	HbA1c < 6.5		HbA1c 6.5–7.9		HbA1c > 8		*F* value	*P* value
Acoustic Reflex	Ipsilateral	*n*	Mean ± SD	*n*	Mean ± SD	*n*	Mean ± SD		
	500 Right	04	88.75 ± 10.31	09	90.56 ± 9.50	28	87.46 ± 16.70	0.147	0.864
Thresholds	1000 Right	04	88.75 ± 10.31	08	91.25 ± 11.57	18	92.50 ± 10.47	0.207	0.814
(dBHL)	2000 Right	04	93.75 ± 13.15	05	86.00 ± 10.84	09	92.78 ± 11.21	0.690	0.517
	BBN Right	05	79.00 ± 14.32	09	80.56 ± 15.09	25	80.80 ± 10.67	0.045	0.956
	500 Left	05	93.00 ± 11.51	09	91.67 ± 11.46	28	92.86 ± 9.17	0.053	0.948
	1000 Left	05	91.00 ± 6.52	09	91.67 ± 7.07	17	93.24 ± 8.47	0.216	0.807
	2000 Left	04	92.50 ± 8.66	05	86.00 ± 4.18	09	95.56 ± 10.74	1.811	0.197
	BBN Left	03	80.00 ± 8.66	08	80.00 ± 7.07	26	85.19 ± 8.06	1.668	0.204

Contralateral	*n*	Mean ± SD	*n*	Mean ± SD	*n*	Mean ± SD	*F* value	*P* value
500 Right	04	92.50 ± 5.00	09	97.22 ± 7.95	28	98.57 ± 7.68	1.149	0.328
1000 Right	03	96.67 ± 5.77	08	98.13 ± 8.84	17	99.12 ± 7.95	0.135	0.874
2000 Right	02	92.50 ± 10.61	03	88.33 ± 5.77	10	97.00 ± 7.53	1.603	0.241
BBN Right	05	85.00 ± 13.23	09	82.22 ± 11.76	25	85.80 ± 7.31	0.494	0.614
500 Left	05	99.00 ± 5.48	09	97.78 ± 9.05	28	99.29 ± 8.36	0.114	0.893
1000 Left	05	97.00 ± 4.47	09	95.00 ± 5.59	16	98.44 ± 6.51	0.954	0.398
2000 Left	04	95.00 ± 7.07	04	90.00 ± 9.13	09	99.44 ± 9.83	1.518	0.253

Acoustic Reflex	Ipsilateral	*n*	Mean ± SD	*n*	Mean ± SD	*n*	Mean ± SD	*F* value	*P* value
	500 Right	05	0.30 ± 0.28	09	0.36 ± 0.30	28	0.30 ± 0.27	0.161	0.852
Amplitude	1000 Right	05	0.26 ± 0.31	07	0.29 ± 0.35	16	0.23 ± 0.25	0.108	0.898
(ml)	2000 Right	02	0.82 ± 0.21	05	0.53 ± 0.44	04	0.28 ± 0.44	1.139	0.367
	BBN Right	05	0.27 ± 0.25	09	0.36 ± 0.26	28	0.23 ± 0.25	0.808	0.453
	500 Left	04	0.47 ± 0.31	09	0.46 ± 0.26	28	0.27 ± 0.27	2.215	0.123
	1000 Left	04	0.27 ± 0.38	09	0.36 ± 0.35	17	0.28 ± 0.34	0.176	0.840
	2000 Left	01	NA	05	0.65 ± 0.37	04	0.43 ± 0.47	0.807	0.484
	BBN Left	05	0.42 ± 0.29	09	0.34 ± 0.30	28	0.17 ± 0.22	3.307	0.057

Contralateral	*n*	Mean ± SD	*n*	Mean ± SD	*n*	Mean ± SD	*F* value	*P* value
500 Right	05	0.34 ± 0.36	09	0.44 ± 0.37	28	0.26 ± 0.26	1.329	0.276
1000 Right	04	0.37 ± 0.33	08	0.28 ± 0.34	17	0.32 ± 0.29	0.122	0.886
2000 Right	02	0.37 ± 0.36	04	0.58 ± 0.38	06	0.25 ± 0.30	1.196	0.346
BBN Right	05	0.41 ± 0.40	09	0.46 ± 0.43	28	0.15 ± 0.23	4.292	0.211
500 Left	03	0.60 ± 0.26	09	0.56 ± 0.27	28	0.35 ± 0.34	1.998	0.150
1000 Left	02	0.09 ± 0.04	09	0.18 ± 0.21	16	0.15 ± 0.20	0.186	0.831
2000 Left	01	NA	05	0.56 ± 0.29	04	0.20 ± 0.31	1.785	0.236
BBN Left	05	0.51 ± 0.41	08	0.40 ± 0.44	28	0.17 ± 0.26	3.481	0.415

Acoustic Reflex	Ipsilateral	*n*	Mean ± SD	*n*	Mean ± SD	*n*	Mean ± SD	*F* value	*P* value
	Right 10%—on 500	05	113.20 ± 54.97	09	131.00 ± 61.72	28	103.64 ± 40.94	1.140	0.330
Latency (ml sec)	Right 10%—off 500	05	164.00 ± 99.94	09	215.22 ± 91.89	28	202.57 ± 72.66	0.681	0.512
	Right 10%—on 1000	05	128.60 ± 89.94	09	166.56 ± 96.29	16	134.88 ± 67.75	0.545	0.586
	Right 10%—off 1000	05	131.80 ± 25.26	08	156.25 ± 19.98	15	148.13 ± 15.79	2.618	0.093
	Right 90%—on 500	05	305.00 ± 76.58	09	304.11 ± 102.56	28	241.43 ± 116.61	1.522	0.231
	Right 90%—off 500	05	372.80 ± 109.50	09	361.78 ± 106.78	28	299.43 ± 84.47	2.433	0.101
	Right 90%—on 1000	05	348.60 ± 69.61	09	327.33 ± 79.52	16	291.88 ± 65.73	1.532	0.234
	Right 90% -off 1000	05	383.60 ± 119.96	08	335.13 ± 75.15	16	274.50 ± 86.85	3.234	0.056
	Left 10%—on 500	05	91.60 ± 38.92	09	117.56 ± 43.88	28	105.00 ± 63.82	0.336	0.717
	Left 10%—off 500	05	152.40 ± 71.95	09	173.56 ± 81.39	28	184.46 ± 82.48	0.350	0.707
	Left 10%—on 1000	05	123.40 ± 34.62	09	129.33 ± 32.54	16	117.88 ± 31.82	0.363	0.699
	Left 10%—off 1000	05	166.00 ± 43.47	09	189.78 ± 38.29	16	168.75 ± 47.60	0.758	0.478
	Left 90%—on 500	05	270.80 ± 49.12	09	287.67 ± 51.05	28	231.43 ± 83.83	2.165	0.128
	Left 90%—off 500	05	362.00 ± 123.98	09	356.106.72	28	285.93 ± 102.68	2.205	0.124
	Left 90%—on 1000	05	357.00 ± 10.05	09	342.67 ± 12.34	16	331.56 ± 56.54	0.715	0.498
	Left 90%—off 1000	05	354.00 ± 121.98	09	404.67 ± 121.68	16	349.69 ± 114.43	0.664	0.523

Contralateral	*n*	Mean ± SD	*n*	Mean ± SD	*n*	Mean ± SD	*F* value	*P* value
Right 10%—on 500	5	126 ± 55.14	9	143.6 ± 68.7	28	117 ± 41.5	0.98	0.3844
Right 10%—off 500	5	176.8 ± 90.66	9	237.1 ± 92.54	28	223.3 ± 73.92	0.96	0.3921
Right 10%—on 1000	5	144 ± 90.76	9	188.3 ± 95.24	16	155.3 ± 74.31	0.61	0.5496
Right 10%—off 1000	5	163.6 ± 36.29	8	177.8 ± 24.41	13	177.3 ± 35.15	0.37	0.6954
Right 90%—on 500	5	348.6 ± 69.61	9	325.4 ± 99.21	28	258.1 ± 123.57	1.46	0.245
Right 90%—off 500	5	406.8 ± 121.56	9	385.4 ± 104.54	28	324.8 ± 87.8	2.47	0.0974
Right 90%—on 1000	5	367 ± 70.82	9	349.7 ± 77.5	16	318.3 ± 67.06	1.14	0.3334
Right 90% -off 1000	5	403.8 ± 127.4	8	348.1 ± 71.34	16	294.8 ± 89.21	2.94	0.0709
Left 10%—on 500	5	118.8 ± 29.91	9	134.3 ± 52.34	28	126.6 ± 67.33	0.11	0.8978
Left 10%—off 500	5	175.2 ± 74.08	9	194.2 ± 81.1	28	205.5 ± 83.32	0.31	0.7321
Left 10%—on 1000	5	145.4 ± 38.08	9	154.6 ± 35.54	16	134.6 ± 35.39	0.92	0.4117
Left 10%—off 1000	5	187.4 ± 47.33	9	210 ± 36.02	16	190.9 ± 50.66	0.59	0.5593
Left 90%—on 500	5	270.4 ± 23.56	9	300.9 ± 48.83	28	259.5 ± 88.11	0.99	0.3823
Left 90%—off 500	5	373.2 ± 124.8	9	379.9 ± 118.38	28	315.4 ± 115.08	1.32	0.2779
Left 90%—on 1000	5	381.2 ± 11.78	9	354.1 ± 31.57	16	345.1 ± 60.56	1.06	0.3613
Left 90%—off 1000	5	375.6 ± 126.24	9	440.2 ± 132.33	16	382.4 ± 125.63	0.69	0.5112

*HbA1c*, Glycosylated haemoglobin; *NA*, Not Available; *SD*, Standard deviation; *F value*, Coefficient of ANOVA

**Table 2:** Subgroup analysis of AR parameters in comparison with duration of DM (ANOVA analysis)

Tests	Variables (Hz)	5–9 years		10–14 years		> = 15 years		*F* value	*P* value
Acoustic Reflex	Ipsilateral	*n*	Mean ± SD	*n*	Mean ± SD	*n*	Mean ± SD		
	500 Right	25	86.96 ± 17.57	14	90.71 ± 7.81	02	87.50 ± 17.68	0.285	0.753
Thresholds	1000 Right	20	91.75 ± 9.77	08	91.88 ± 11.32	02	90.00 ± 21.21	0.026	0.975
(dBHL)	2000 Right	14	93.21 ± 11.02	03	86.67 ± 10.41	01	NA	1.589	0.237
	BBN Right	23	81.74 ± 11.44	14	80.36 ± 11.84	02	67.50 ± 17.68	1.341	0.274
	500 Left	26	92.50 ± 8.63	14	93.57 ± 11.17	02	87.50 ± 17.68	0.336	0.716
	1000 Left	22	92.73 ± 6.86	07	91.43 ± 8.52	02	92.50 ± 17.68	0.072	0.930
	2000 Left	15	94.00 ± 9.10	02	85.00 ± 7.07	01	NA	1.866	0.189
	BBN Left	21	82.86 ± 6.24	14	85.71 ± 9.97	02	77.50 ± 10.61	1.156	0.327

Contralateral	*n*	Mean ± SD	*n*	Mean ± SD	*n*	Mean ± SD	*F* value	*P* value
500 Right	25	98.80 ± 7.54	14	95.36 ± 6.92	02	100.00 ± 14.14	1.002	0.370
1000 Right	18	99.17 ± 6.00	08	95.63 ± 7.76	02	105.00 ± 21.21	1.335	0.281
2000 Right	11	95.91 ± 8.01	03	93.33 ± 7.64	01	NA	0.917	0.426
BBN Right	23	86.30 ± 9.07	14	83.57 ± 8.19	02	77.50 ± 17.68	1.079	0.351
500 Left	26	99.81 ± 7.94	14	97.50 ± 7.54	02	97.50 ± 17.68	0.392	0.678
1000 Left	21	96.67 ± 5.55	07	98.57 ± 6.90	02	97.50 ± 10.61	0.257	0.776
2000 Left BBN Left	14 24	97.86 ± 9.35 85.42 ± 11.51	02 14	90.00 ± 7.07 83.93 ± 10.03	01 02	NA 77.50 ± 24.75	1.421 0.457	0.274 0.637

Acoustic Reflex	Ipsilateral	*n*	Mean ± SD	*n*	Mean ± SD	*n*	Mean ± SD	*F* value	*P* value
	500 Right	26	0.28 ± 0.26	14	0.31 ± 0.28	02	0.67 ± 0.21	1.958	0.155
Amplitude	1000 Right	19	0.27 ± 0.28	07	0.13 ± 0.18	02	0.43 ± 0.52	1.184	0.323
(ml)	2000 Right	08	0.55 ± 0.41	02	0.06 ± 0.00	01	NA	2.003	0.197
	BBN Right	26	0.26 ± 0.25	14	0.27 ± 0.27	02	0.31 ± 0.35	0.042	0.959
	500 Left	25	0.33 ± 0.26	14	0.33 ± 0.30	02	0.41 ± 0.47	0.068	0.935
	1000 Left	21	0.32 ± 0.33	07	0.21 ± 0.34	02	0.44 ± 0.57	0.426	0.657
	2000 Left	07	0.69 ± 0.33	02	0.04 ± 0.00	01	NA	4.491	0.056
	BBN Left	26	0.20 ± 0.22	14	0.28 ± 0.32	02	0.30 ± 0.34	0.467	0.630

Contralateral	*n*	Mean ± SD	*n*	Mean ± SD	*n*	Mean ± SD	*F* value	*P* value
500 Right	26	0.29 ± 0.30	14	0.28 ± 0.28	02	0.66 ± 0.40	1.479	0.240
1000 Right	20	0.29 ± 0.28	07	0.46 ± 0.38	02	0.10 ± 0.02	1.412	0.262
2000 Right	09	0.42 ± 0.35	02	0.06 ± 0.01	01	NA	1.269	0.327
BBN Right	26	0.24 ± 0.32	14	0.22 ± 0.30	02	0.52 ± 0.65	0.761	0.474
500 Left	24	0.43 ± 0.31	14	0.37 ± 0.35	02	0.48 ± 0.60	0.191	0.827
1000 Left	18	0.16 ± 0.17	07	0.17 ± 0.27	02	0.09 ± 0.03	0.135	0.874
2000 Left	07	0.50 ± 0.31	02	0.04 ± 0.00	01	NA	2.331	0.168
BBN Left	25	0.25 ± .032	14	0.23 ± 0.34	02	0.50 ± 0.61	0.541	0.586

Acoustic Reflex	Ipsilateral	*n*	Mean ± SD	*n*	Mean ± SD	*n*	Mean ± SD	*F* value	*P* value
	Right 10%—on 500	26	113.96 ± 50.75	14	98.93 ± 33.99	02	149.50 ± 91.22	1.162	0.324
Latency	Right 10%—off 500	26	196.96 ± 86.68	14	198.71 ± 62.18	02	263.00 ± 111.72	0.637	0.534
(ml sec)	Right 10%—on 1000	21	144.29 ± 82.73	07	125.14 ± 54.59	02	197.00 ± 140.01	0.627	0.542
	Right 10%—off 1000	20	144.25 ± 20.41	06	156.33 ± 20.02	02	154.00 ± 2.83	0.962	0.396
	Right 90%—on 500	26	262.00 ± 114.02	14	248.29 ± 111.23	02	367.00 ± 35.36	0.988	0.382
	Right 90%—off 500	26	336.23 ± 102.17	14	289.43 ± 74.36	02	355.00 ± 134.35	1.238	0.301
	Right 90%—on 1000	21	305.29 ± 79.76	07	323.71 ± 37.85	02	341.00 ± 103.24	0.331	0.721
	Right 90% -off 1000	20	321.50 ± 93.43	07	273.14 ± 96.64	02	324.50 ± 167.58	0.654	0.528
	Left 10%—on 500	26	101.46 ± 55.54	14	114.29 ± 64.89	02	109.00 ± 18.38	0.223	0.801
	Left 10%—off 500	26	176.12 ± 90.90	14	175.71 ± 58.99	02	225.00 ± 74.95	0.394	0.678
	Left 10%—on 1000	21	125.67 ± 34.34	07	114.57 ± 26.95	02	113.00 ± 21.21	0.180	0.836
	Left 10%—off 1000	21	174.10 ± 43.40	07	172.29 ± 48.24	02	188.00 ± 65.05	0.163	0.851
	Left 90%—on 500	26	249.46 ± 80.14	14	241.71 ± 74.40	02	276.50 ± 99.70	0.347	0.709
	Left 90%—off 500	26	323.00 ± 109.87	14	279.07 ± 105.48	02	361.00 ± 131.52	0.097	0.908
	Left 90%—on 1000	21	336.90 ± 46.29	07	347.29 ± 35.94	02	334.00 ± 31.11	0.967	0.389
	Left 90%—off 1000	21	369.19 ± 117.71	07	347.14 ± 112.56	02	412.00 ± 181.02	0.242	0.787

Contralateral	*n*	Mean ± SD	*n*	Mean ± SD	*n*	Mean ± SD	*F* value	*P* value
Right 10%—on 500	26	126.1 ± 52.57	14	113.1 ± 34.2	2	167.5 ± 106.77	1.14	0.3318
Right 10%—off 500	26	216 ± 87.99	14	220.3 ± 59.46	2	285 ± 113.14	0.68	0.5103
Right 10%—on 1000	21	164.8 ± 85.6	7	145 ± 60.54	2	212 ± 145.66	0.51	0.6077
Right 10%—off 1000	19	168.4 ± 26.78	5	195.4 ± 47.47	2	184 ± 7.07	1.6	0.2229
Right 90%—on 500	26	282.1 ± 110.76	14	258.7 ± 128.34	2	390 ± 28.28	1.15	0.3285
Right 90%—off 500	26	361.9 ± 106.51	14	316.5 ± 77.91	2	378.5 ± 127.99	1.07	0.3535
Right 90%—on 1000	21	329.8 ± 79.32	7	347.1 ± 37.64	2	359.5 ± 99.7	0.26	0.7731
Right 90% -off 1000	20	343.9 ± 95.73	7	289.1 ± 102.31	2	309.5 ± 127.99	0.84	0.4451
Left 10%—on 500	26	122.6 ± 57.44	14	137.5 ± 69.96	2	117.5 ± 19.09	0.3	0.7459
Left 10%—off 500	26	197.4 ± 92.44	14	196.9 ± 57.56	2	245.5 ± 71.42	0.33	0.7201
Left 10%—on 1000	21	148.2 ± 38	7	131.4 ± 28.87	2	119 ± 22.63	1.04	0.3667
Left 10%—off 1000	21	195.1 ± 43.69	7	194.3 ± 54.52	2	212.5 ± 61.52	0.13	0.8774
Left 90%—on 500	26	267 ± 77.96	14	269.8 ± 77.24	2	303 ± 103.24	0.2	0.8236
Left 90%—off 500	26	342 ± 112.54	14	318.1 ± 128.45	2	385.5 ± 163.34	0.36	0.6999
Left 90%—on 1000	21	352.9 ± 51.88	7	370.4 ± 34.55	2	305 ± 28.28	1.47	0.2484
Left 90%—off 1000	21	398 ± 126.63	7	384.1 ± 128.18	2	455 ± 189.5	0.23	0.7943

*NA*, Not Available; *SD*, Standard deviation; *F value*, Coefficient of ANOVA

## Discussion

In the present study, mean PTA was comparatively greater in the cases than in controls although all the subjects included in the study had normal hearing thresholds (PTA < 25 dBHL) which demonstrates the positive association between T2DM and hearing loss. Kim MB et al. conducted a large cohort study which showed the development of bilateral hearing loss in diabetics [9]. A similar study was done by Dosemane et al., which postulated that bilateral SNHL is a complication of T2DM [4]. Akinpelu et al. conducted a meta-analysis and reviewed 18 articles which revealed an increased incidence of HL ranging from 44 to 69.7% and prolonged auditory brainstem response (ABR) wave V latencies in the subjects with T2DM which may be due to degeneration of hair cells in basal turn of cochlea and delay in the conduction of auditory signals within the brain stem with diabetes, respectively [10]. Another meta-analysis has been conducted by Mujica-Mota et al. which showed raised incidence of HL, lower oto-acoustic emissions (OAE) and prolonged latencies in ABR waves I, III and V in type 1 diabetes mellitus (T1DM) subjects [11]. In the present study, no significant difference was found in SIS between cases and controls. A study conducted by Huang et al. revealed significant decrease in SDS scores in diabetics which correlated with the high-frequency sensorineural hearing loss [12].

A study was done to assess the ART and RDT in the geriatric group by Ünsal et al. who concluded that although some changes were observed due to age, middle ear and stapedius work normally in geriatric category as no significant difference was found between geriatric and non-geriatric categories in ipsilateral and contralateral AR parameters and RDT [13]. Virtaniemi et al. concluded that decreased ARAs and prolonged ARLs in subjects with insulin-dependent diabetes mellitus (IDDM) were more probably attributed to the rigid middle ear structure than brainstem alterations [14]. Another similar study was done by Braite et al. who observed the absence of an inhibitory effect of medial olivocochlear reflex (MOC) with distortion product OAE (DPOAE) at 4000, 6000 and 8000 Hz in patients with T1DM as a result of early auditory dysfunction of the efferent pathway [6]. To the best of our understanding, no prior research has reported AR parameters in patients with T2DM. In the present study, we found a significant difference in ARA (decreased amplitudes at lower frequencies and BBN) and ARL in cases when compared to controls which may be indicative of damage to the neural auditory pathway. However, there was no clear explanation for the significant difference between the ipsilateral right and left ARL. There was no evidence of significant difference in the contralateral right and left ARL.

Our study showed no significant association found between AR parameters and other variables such as duration of DM and control of DM based on HbA1c levels. However, literature shows discrepancies; Mujica-Mota et al. demonstrated an increased risk of HL over time with an increase of prevalence by 1.7% per 1-year exposure of DM and another study conducted by Mishra et al. revealed increased severity of SNHL with duration of DM [11, 15]. On the contrary, analysis conducted by Kim et al. interestingly showed a stronger association of HL in the younger group (< 50 years) [9]. Various other investigators observed no association of HL with duration of DM [4, 6, 16, 17]. A positive correlation was noted between the severity of DM and degree of HL in a study by Mishra et al.; profound SNHL was highly prevalent in diabetics with FBS > 200 mg/dL [15]. Srinivas et al. evaluated the association between poorly controlled DM (HbA1c > 8) with SNHL; the prevalence of SNHL is more than 85% in the subjects with poor glycaemic control and duration of DM of more than 10 years [18]. Our study was unique as confounding factors known to cause HL were eliminated due to exclusion criteria and objective tests have been used to assess the effect of T2DM on the auditory pathway.

## Conclusion

T2DM can lead to increased hearing thresholds, decreased ARA at lower frequencies and BBN. Hence, evaluating AR parameters in patients with T2DM may help detect the early effects of DM on the auditory pathway. However, there is no association between independent variables such as duration and glycaemic control of T2DM.

## Data Availability

Data transparent.

## References

[1] Giraudet F, Mulliez A, de Resende LM, Beaud L, Benichou T, Brusseau V (2022). Impaired auditory neural performance, another dimension of hearing loss in type-2 diabetes mellitus. Diabetes & Metabolism.

[2] Fukushima H, Cureoglu S, Schachern P, Paparella M, Harada T, Oktay M (2006). Effects of Type 2 Diabetes Mellitus on Cochlear Structure in Humans. Arch Otolaryngol Head neck Surg.

[3] Bainbridge KE, Hoffman HJ, Cowie CC (2008). Diabetes and hearing impairment in the United States: audiometric evidence from the National Health and Nutrition Examination Survey, 1999 to 2004. Ann Intern Med.

[4] Dosemane D, Bahniwal RK, Manisha N, Khadilkar MN (2019). Association Between Type 2 Diabetes Mellitus and Hearing Loss Among Patients in a Coastal City of South India. Indian J Otolaryngol Head Neck Surg.

[5] Cichosz SL, Hejlesen O (2018). Association between diabetes and changes in hearing: A cross-sectional study. Diabetes Metab.

[6] Braite N, da Cruz Fernandes L, Rissatto Lago MR, de Arag ão Dantas Alves C (2019) Effects of type 1 diabetes mellitus on efferent auditory system in children and adolescents. Int J Pediatr Otorhinolaryngol 127:109660. 10.1016/j.ijporl.2019.109660 10.1016/j.ijporl.2019.109660 31487561

[7] Canale A, Albera R, Lacilla M, Canosa A, Albera A, Sacco F, Chiò A, Calvo A (2017). Acoustic reflex patterns in amyotrophic lateral sclerosis. Eur Arch Otorhinolaryngol.

[8] Narayanan R. (2017). Characterization of acoustic reflex latency in females. Global Journal of Otolaryngology, 11(2). 10.19080/gjo.2017.11.555808

[9] Kim MB, Zhang Y, Chang Y, Ryu S, Choi Y, Kwon MJ (2017). Diabetes mellitus and the incidence of hearing loss : a cohort study. Int J Epidemiol.

[10] Akinpelu OV, Mujica-Mota M, Daniel SJ (2014). Is type 2 diabetes mellitus associated with alterations in hearing? A systematic review and meta-analysis. Laryngoscope.

[11] Mujica-mota MA, Patel N, Saliba I (2018). Hearing loss in type 1 diabetes : Are we facing another microvascular disease ? A meta-analysis. Int J Pediatr Otorhinolaryngol.

[12] Huang YM, Pan CY, Gu R, Cai XH, Yu LM, Qiu CY (1992). Hearing impairment in diabetics. Chin Med J.

[13] Ünsal S, Karataş H, Kaya M, Gümüş NM, Temügan E, Yüksel M (2016). Evaluation of Acoustic Reflex and Reflex Decay Tests in Geriatric Group. Turk Arch Otorhinolaryngol.

[14] Virtaniemi J, Laakso M, Nuutinen J, Karjalainen S, Vartiainen E (1994). Acoustic-Reflex Responses in Patients With Insulin-Dependent Diabetes Mellitus. Am J Otolaryngol.

[15] Mishra A, Poorey VK (2019). Clinical and Audiometric Assessment of Hearing Loss in Diabetes Mellitus. Indian J Otolaryngol Head Neck Surg.

[16] Gupta S, Eavey RD, Wang M, Curhan SG, Curhan GC (2019). Type 2 diabetes and the risk of incident hearing loss. Diabetologia.

[17] Mitchell P, Gopinath B, McMahon CM, Rochtchina E, Wang JJ, Boyages SC (2009). Relationship of Type 2 diabetes to the prevalence, incidence and progression of age-related hearing loss. Diabet Med.

[18] Srinivas CV, Shyamala V, Shiva Kumar BR (2016). Clinical Study to Evaluate the Association Between Sensorineural Hearing Loss and Diabetes Mellitus in Poorly Controlled Patients Whose HbA1c >8. Indian J Otolaryngol Head Neck Surg.

